# Exploring the Socio‐Economic Toll of Non‐Communicable Diseases in Low‐Resource Settings: Novel Evidence From Mozambique

**DOI:** 10.1002/hpm.70091

**Published:** 2026-05-19

**Authors:** Maria Verykiou, Paolo Belardi, Sandra Loureiro, Paolo Massaro, Fernando Chenene, Celina Mate, Celeste Amado, Armindo Daniel Tiago, Giovanni Putoto, Aleksandra Torbica

**Affiliations:** ^1^ SDA Bocconi School of Management Milan Italy; ^2^ Doctors with Africa CUAMM Padova Italy; ^3^ Mozambican Diabetes Association (AMODIA) Maputo Mozambique; ^4^ Doctors with Africa CUAMM Maputo Mozambique; ^5^ Ministry of Health of Mozambique (MISAU) Maputo Mozambique

**Keywords:** costs, diabetes, economic burden, hypertension, low‐income countries, non‐communicable diseases

## Abstract

**Background:**

The socio‐economic burden of increasingly prevalent non‐communicable diseases (NCDs) in low‐income countries is substantial and widely recognised. This study aimed to fill the remaining evidence gap for type 2 diabetes (T2DM) and hypertension (HTN) patients in selected health facilities across two provinces in Mozambique.

**Methods:**

A cross‐sectional cost‐of‐illness study was conducted measuring the societal costs incurred by patients enroled in an integrated disease management programme. Direct healthcare and non‐healthcare costs, and indirect costs, were reported in current US$ ($) values per month per patient. The dimensions across which the costs were analysed from an equity lens were disease groups, location, sex, and income groups using the World Bank's poverty line thresholds for Mozambique.

**Results:**

Programme costs amounted to $1.2 M ($3.2 per patient visited), while the recurrent costs averaged $49.8 per month per patient, with direct non‐healthcare costs as the main driver at 74% of the total recurrent costs. Higher costs across all cost categories were consistently reported by T2DM patients, males, and patients from the upper‐income group. Higher direct but lower indirect costs were reported by patients from rural rather than urban locations. The costs of the lowest‐income group accounted for the majority of their reported monthly income, while 3% of the sample reported that their income is insufficient to cover the costs of managing their disease.

**Conclusions:**

The socio‐economic burden was disproportionately higher in the lowest income group. The study highlighted the need for policies and interventions such as targeting the financing of managing chronic diseases, including potential increases in funding, the introduction of patient cost‐sharing mechanisms, and effective intersectoral coordination to mitigate economic strain in low‐income settings.

## Highlights

- Study offers first comprehensive economic analysis of NCDs in Mozambique.
- NCDs strain Mozambique's healthcare with costs 25% above average expenditure.
- Costs for poor patients highlight severe inequities in Mozambique's NCD impact.
- LIC healthcare systems need innovative funding to manage chronic disease costs. Findings urge policymakers to boost funding for sustainable NCD management.

## 1 Introduction

Today, Non‐Communicable Diseases (NCDs) affect Low‐Income Countries (LIC) more than High Income Countries (HIC), with 77% of all NCD deaths coming from LICs, and this trend is expected to worsen in the foreseeable future [1]. According to the World Health Organisation (WHO), the number of people living with diabetes rose to 830 million worldwide by 2022, with the majority living in Low‐and Middle‐Income Countries (LMIC), while it constitutes the ninth leading cause of death globally with 1.5 million deaths reported annually [1, 2, 3]. On the other hand, hypertension (HTN) accounts for 12.8% of mortality globally [4]. Approximately 95% of the diabetes cases are attributed to Type 2 diabetes mellitus (T2DM), with patients twice as likely to suffer from HTN compared to those without diabetes, contributing significantly to morbidity in diabetic patients due to cardiovascular diseases [5]. Both diseases, T2DM and HTN, have demonstrated rising trends in prevalence, and cases are expected to continue increasing significantly by 2040 [2, 3, 6], respectively. In Sub‐Saharan Africa, diabetes is expected to rise to 40.7 million people by 2045 [7].

Moreover, diabetes and HTN are of economic importance due to the high costs associated with the continuous monitoring of the diseases and the costs of treating complications [8, 9, 10]. As chronic diseases, they can potentially present an additional economic burden to households, who may experience productivity losses and additional costs to accommodate for lifestyle changes of the patients affected [11, 12]. Such burden can lead to catastrophic costs, especially in settings where high out‐of‐pocket (OOP) expenditures are associated with covering medical expenses, such as in LMIC [13, 14, 15].

Based on WHO data as of 2016 [16], in Mozambique's 27,978,000 population, the diabetes prevalence in adults aged between 20 and 79 was equal to 2.4% [17], and a total number of 3220 people die annually of diabetes, representing 1% of annual mortality in the country. For HTN, Mozambique is included in the WHO's list of top 10 countries with the lowest treatment rate in 2019 both for men and women [18]. Indeed, while the global trend of HTN prevalence is going down, in Mozambique the rate for both men and women is increasing and is currently at 23% of the total population [19].

In terms of policy response, in 2007, the First National Strategic Plan for the Prevention and Control of NCD 2008–2014 was drawn up (later extended until 2019) with the aim of reducing risk factors and guaranteeing increased access to health for chronic patients [20, 21]. Among the measures adopted, hypertensive and anti‐glycaemic drugs were included in the list of essential medicines. Under the Strategic Plan 2020–2029, the Ministry of Health of Mozambique (MISAU) has renewed its commitment to achieve 10% reduction in the prevalence of HTN in the country and to maintain the prevalence of diabetes unchanged by 2029, focussing on primary prevention and strengthening care at the community level [22]. Despite MISAU's efforts, the implementation of these plans faces chronic resource shortages: public health expenditure remains constrained—averaging approximately US$ 52.96 per capita as of 2023 [23], resulting in the shortage of qualified healthcare personnel, inadequate infrastructure, and geographical disparities in access to health services [24, 25, 26]. Additionally, a significant portion of the NCD‐dedicated budget in Mozambique was traditionally absorbed by tertiary‐level hospital care, creating a chronic funding gap at the primary and secondary levels, where the management of T2DM and HTN is most critical [22, 24, 27].

A desk review of published literature on the Cost‐of‐Illness (COI) of HTN and T2DM in Sub‐Saharan Africa revealed that studies variably adopted patient/household [8, 10, 12, 28, 29], health system/provider [30, 31], or societal perspectives [30, 32, 33, 34].

Studies included different combinations of costs, typically categorised into direct medical costs [29, 35, 36], direct non‐medical costs, such as transportation and diet [37, 38], and indirect costs related to productivity losses and to waiting and travel times [10, 32, 39, 40]. The identified recurrent cost drivers were medications, inpatient or complication‐related care, diagnostics, transport, and productivity losses, while the risk of catastrophic expenditure was greatest among poorer and more vulnerable groups in many country‐specific studies [10, 12, 28, 30, 38].

Systematic reviews highlighted the scarcity of representative estimates at the national level, the predominance of single‐country studies, and the lack of methodological standardisation, all of which limit direct cross‐study comparability [35, 40, 41, 42, 43, 44, 45]. For T2DM, studies underscored the importance of medicines and complication‐related care as major drivers of expenditure, while showing that amounts varied widely across different settings [35, 43, 46]. Afroz et al. reported annual per‐person total costs of T2DM ranging from US$29.91 to US$237.38, direct costs from US$106.53 to US$293.79, and indirect costs from US$1.92 to US$73.4 [43]. Moucheraud et al. similarly estimated wide ranges in T2DM costs, with outpatient costs ranging from under US$5 to over US$40, annual inpatient costs from about US$10 to more than US$1,000, annual laboratory costs from under US$5 to over US$100, and annual medication costs from US$15 to over US$500 [46]. A more recent systematic review reported annual per‐patient diabetes costs ranging from US$87 to US$9581 and with direct medical costs accounting for roughly 56% of total costs in LMICs and 74% in HICs, while inpatient care and medicines emerged as the largest components of expenditure [35]. For HTN, Gnugesser et al. identified 33 eligible studies representing one quarter of the countries in the region and found that medication costs were the most prominent and recurrent component from both the patient and provider perspectives [40]. The review further highlighted multidrug regimens, inpatient care, comorbidities such as diabetes, distance to care, private‐sector use, and hospital‐based treatment as major cost drivers, while catastrophic health expenditure was especially severe among poorer, rural, and less educated patients [40]. These findings were consistent with a broader regional review, which highlighted how low detection and treatment rates, health‐system constraints, and poor access to affordable long‐term care amplified the economic consequences of HTN [45].

Individual country‐level studies provided more granular evidence of the economic burden and the specific cost drivers of HTN and T2DM. In Nigeria, research on T2DM revealed an annual economic burden of approximately US$351.50 per patient, where medications alone accounted for nearly 40% of direct costs. Notably, 45.1% of these households faced catastrophic health expenditure [36]. Evidence from Mali emphasised the financial impact of diabetes‐related complications, with total 90‐day costs nearly four times higher than for non‐diabetic individuals (US$281.92 vs. US$77.08) [39]. A recent study in Northern Ghana reported a mean annual direct healthcare cost of US$159.70 for T2DM care, with medication identified as the cost driver (44.8%), while 25% of patients indicated the need to borrow money and at least 21% of patients the need to sell physical assets to cover treatment costs [38]. In Ethiopia, the societal cost of HTN was estimated at US$105.55 per month, with 80.4% of it attributed to indirect costs, primarily driven by productivity losses from premature mortality and morbidity [32]. A study on HTN care in Kenyan public facilities reported a mean annual direct cost of US$304.8 per patient, with medicines (US$168.9) and transport (US$126.7) identified as the primary cost drivers [10].

To the best of our knowledge, none of the evidence found and synthesised above includes information on the economic burden of T2DM or HTN specifically in Mozambique.

Given such premises, the primary objective of this study was to estimate the economic burden of patients affected by T2DM and HTN in selected health facilities from two provinces of Mozambique. As a secondary objective, the study explored differences in costs across different care settings (urban vs. rural facilities), sex, as well as different income groups.

## 2 Methods

### 2.1 Study Design and Setting

The study was designed as a cost‐of‐illness analysis of HTN and T2DM, an established method in health economics to estimate the economic burden of diseases from a societal perspective [47, 48]. The study was conducted in six Health Facilities (HF), four rural and two urban, in two provinces of Mozambique, Maputo and Sofala (see Supporting Information S1: Supporting Information 1).

### 2.2 The NCDs Programme

In 2019, Doctors with Africa CUAMM, ‘Collegio Universitario Aspiranti Medici Missionari’ (CUAMM), an International Non‐Governmental Organisation (INGO) focused on local health systems strengthening in Sub‐Saharan Africa, implemented a disease management intervention for HTN and T2DM. This programme was jointly identified and agreed upon by the Ministry of Health of Mozambique (MISAU) and AICS (Italian Cooperation Agency, Ministry Foreign Officer Affairs). CUAMM was the leading agency for the implementation along with other Italian NGOs and local health authorities.

The programme introduced resources; equipment, laboratory reagents, drugs and consumables, and materials needed for the diagnosis, treatment, and monitoring of patients with T2DM, HTN, and patients with both pathologies and included a capacity building component of the healthcare personnel on screening and treatment for these diseases. Finally, the programme introduced updated registers for outpatient visits (i.e., consultas externas) and of medical records (‘fichas’) to support patient monitoring and data management in collaboration with other implementing partners.

### 2.3 Data Collection and Study Population

Both primary as well as secondary data were collected. Sources of primary data included anonymous questionnaires and interviews with patients and healthcare personnel, as well as anonymous extraction of data from randomly selected patient records (‘fichas’) on medical treatment costs. On the other hand, secondary data were available from the programme's archives and project‐related documentation such as reports, budgets, as well as official reporting and documentation sources at the HFs pertaining to accounting data, salaries, cost of equipment, and specific activities.

The study population included patients with T2DM, HTN, and patients with both diseases under the programme. All patients affected by the chronic diseases under investigation were included in the scope of the study (T2DM, HTN, or both). Patients that were subject to hospitalisation were excluded as data collection at the secondary level of care was not implemented.

A sampling process for the selection of HFs visited for data collection was followed starting from the total HFs in which the intervention had been rolled out. HFs where the intervention was not implemented and HFs with a very low number of patient visits for T2DM, defined as equal or less than 5 patient visits per month, were excluded (see Supporting Information S1: Supporting Information 2). The final sample size contained 3 HFs per Province with a total of 6 HFs, 4 Rural (Moamba Sede, Nhaconjo, Sao Damaso, Nhamatanda) and 2 Urban (Macarungo, Matola). In total, 298 patient questionnaires, 100 from Maputo and 198 from Sofala [1], were completed, (Supporting Information S1: Supporting Information 3). Responses were obtained using Kobo Collect through interviews performed by trained data collectors with healthcare workers as well as with patients under informed consent during the month of March 2023. The patient characteristics of the sample as well as the patient pathway are described in the Supporting Information S1: Supporting Information 4 and 5.

### 2.4 Data Analysis

Where respondents provided information on their income, this was used to classify patients in income categories as per the World Bank defined income classifications for Mozambique [49]. For the data analysis and reporting, respondents were classified in groups as under the poverty line (< PL), lower‐middle income (LMI), upper‐middle income (UMI) and upper income (UI). As per the World Bank using $2017 Purchasing power parity (PPP), the poverty line in Mozambique is defined below $2.15, LMI class is defined between $2.15–3.65, UMI class is defined between $3.65–6.85, and UI class is defined as above $6.85 per day.

Costs were initially reported in Mozambican Metical (MTN) and subsequently converted to US dollars ($) using the most recent World Bank exchange rate ($1 = MTN 63.89 in 2023). Data analysis employed a mixed‐methods approach, incorporating both bottom‐up and top‐down techniques, and costs were adjusted to 2023 values using the World Bank's GDP deflator index. Estimates in this paper are presented in 2023 US$ ($) alongside confidence intervals calculated at the 95% confidence level, with significance of estimate differences across groups assessed at the 0.05 significance level using ANOVA and *t*‐test methods. A concentration index was also generated for the income group analysis, based on the distribution of patients across quartiles as per the monthly income reported by them in the questionnaires.

The costs examined included programme implementation costs at the aggregate level and patient‐level costs at the individual level. Total patient costs were categorised into direct healthcare costs (e.g., diagnostics, medical visits, medical equipment, treatments) and direct non‐healthcare costs (e.g., transportation to care facilities, accommodation, nutritional supplements), as well as productivity losses. Direct healthcare costs covered by the healthcare system, such as personnel costs and portions of treatment costs, represented expenses borne by the health system, while other categories reflected the financial burden on patients and their families in managing the chronic diseases. The human capital approach was applied to monetise time costs, using patients' self‐reported monthly incomes and salaries, along with healthcare personnel salaries as outlined in ‘Imprensa Nacional de Mocambique, E.P., Boletim da República, 1^a^ Série—Número 198, 14 October 2022’ [50]. To estimate the implementation cost of the programme at the patient level, the total cost of the programme's implementation, was divided by the cumulative number of visits until March 2022 for each disease. In the cost calculations, it was assumed that the reported visit frequency reported by the patients corresponded to 100% adherence to the programme.

## 3 Results

### 3.1 Health System Costs

**FIGURE 1:**
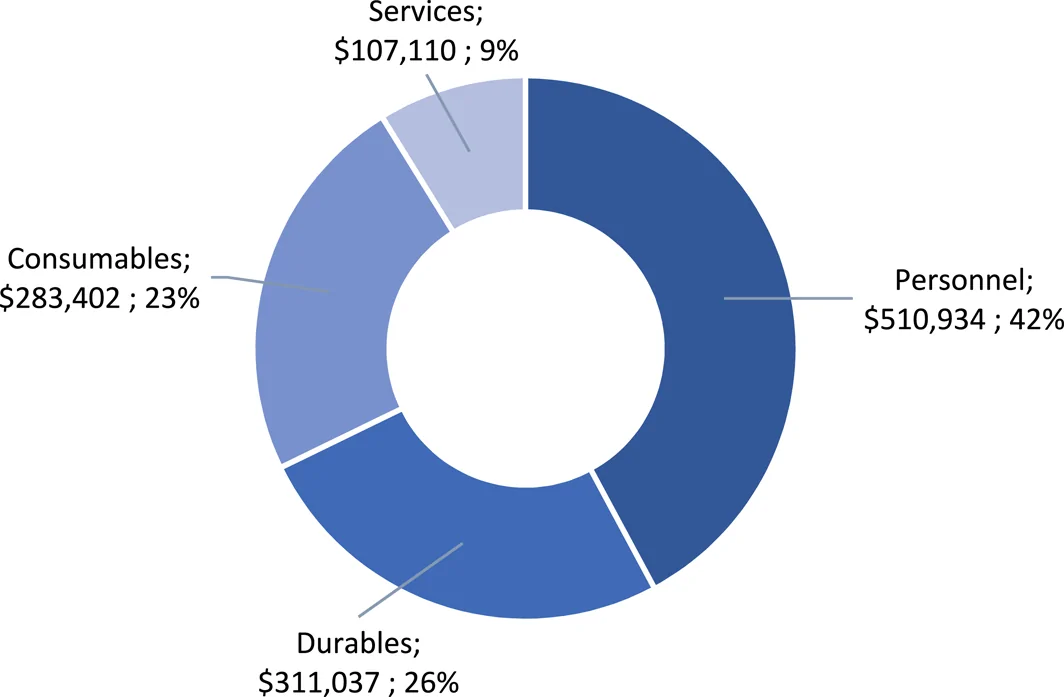
Total programme implementation costs, 2019 ($).

The overall costs incurred for the implementation of the programme in 2019, during its first year of operation, for T2DM and HTN were estimated at $1,212,484. This estimate included personnel, durable goods, consumable goods, and services (Figure 1).

Personnel costs were identified as the major cost category accounting for 42% of the total implementation cost, and pertained to the payment of salaries, daily allowances and travel costs of national staff, such as project managers, coordinators, and doctors (60%) as well as international staff (40%). Durable goods accounted for 26% of the total implementation costs, half of which pertained to the purchasing of vehicles for transportation needs, and the rest for IT, medical and non‐medical equipment. Similarly, consumable goods accounted for 23% of the implementation costs, with the majority of expenditures pertaining to medicines and Covid‐19 related measures. Finally, services accounted for 10% of the implementation costs, driven by costs for supervision activities, trainings and maintenance of offices.

The average cost per visit of the programme's implementation at the patient level considering the total number of visits (385,945) in the programme was estimated at $3.2.

### 3.2 Patient Level Costs

**FIGURE 2:**
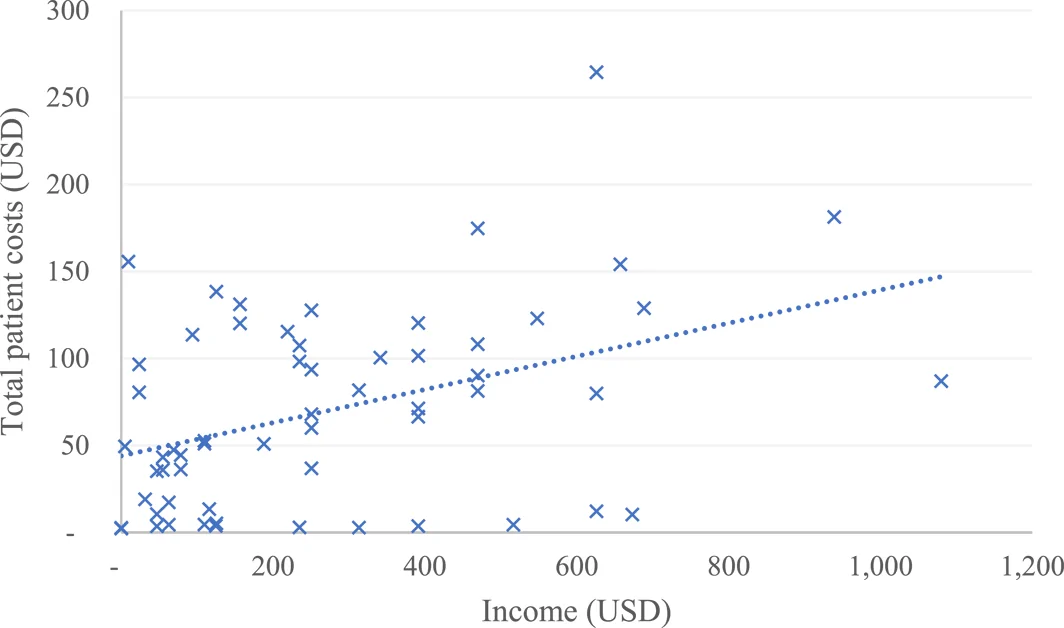
Correlation between monthly income and monthly total patient costs (*r* = 0.47), irrespective of disease group.

The total patient costs per month are presented in Table 1, categorised by cost component per patient disease group, sex, geographic location, and income group. The average total monthly cost per patient was $49.8, with the largest portion (74%) attributed to direct non‐healthcare costs, which amounted to $36.9. Direct healthcare costs and indirect costs from productivity losses were estimated at $8.7 and $4.2 per patient, respectively. Among disease groups, patients with Type 2 Diabetes Mellitus (T2DM) had the highest costs ($62.4), primarily driven by non‐healthcare expenses, followed by those with both HTN and T2DM ($52.2). HTN patients alone incurred significantly lower costs at $35.8. Additionally, male patients reported higher costs across all categories compared to female patients, however, these differences were not significant. In the same line, geographic differences were minimal and non‐significant, though rural patients experienced slightly higher overall costs, with lower productivity losses in rural health facilities ($2.9) compared to urban ones ($5.1). Finally, costs increased with higher income, reflecting a positive correlation between monthly salary and total patient costs (Figure 2). A correlation analysis was performed and reported in Supporting Information S1: Table S2.2 in Supporting Information 5. With respect to the differences of costs incurred across income groups, these were found to be significant at the *p* = 0.05 level with comparable concentration indices of total monthly costs relative to the monthly income reported by patients (CI between 0.32 and 0.49).

**TABLE 1:** Total monthly costs per patient in $ by disease, sex, location, and income group, broken down by type (direct healthcare, direct non‐healthcare and indirect costs), including *p*‐values with significance at *p* < 0.05, as well as correlation index noted for estimates per income group.

	Mean total costs per patient per month (95% CI)			
	Total costs ($)	Direct healthcare costs ($)	Direct non‐healthcare costs ($)	Indirect costs: Productivity losses ($)
Overall	49.8 (45.1; 54.5)	8.7 (7.5; 10)	36.9 (32.6; 41.3)	4.2 (2.9; 5.4)
By disease				
T2DM (*n* = 97)	62.4 (53.3; 71.5)	7.7 (5.2; 10.2)	50.1 (41.6; 58.5)	4.6 (2.4; 6.8)
HTN (*n* = 105)	35.8 (29.3; 42.2)	7.2 (6.2; 8.3)	24.2 (18.3; 30.1)	4.4 (1.9; 6.8)
Both (*n* = 96)	52.2 (44.1; 60.4)	11.4 (9; 13.7)	37.5 (29.9; 45.2)	3.3 (1.6; 5)
*p*‐value	< 0.05	< 0.05	< 0.05	0.69
By sex				
Female (*n* = 197)	45.1 (39.4; 50.7)	8.1 (6.8; 9.3)	33.7 (28.5; 39)	3.3 (1.7; 4.8)
Male (*n* = 101)	58.5 (50.1; 66.8)	10 (7.5; 12.5)	43.1 (35.4; 50.8)	5.4 (3.3; 7.4)
*p*‐value	< 0.05	0.16	0.05	0.11
By location				
Rural (*n* = 138)	49.8 (43.6; 55.9)	9.1 (7.4; 10.8)	37.7 (31.9; 43.5)	2.9 (2; 3.9)
Urban (*n* = 160)	49.7 (42.7; 56.6)	8.4 (6.7; 10.1)	36.2 (29.8; 42.7)	5.1 (3; 7.1)
*p*‐value	0.85	0.49	0.74	0.1
By income group				
< PL (*n* = 42)	41.2 (32.7; 49.7)	7.1 (4.7; 9.6)	33 (24.8; 41.1)	1 (0.5; 1.5)
LMI (*n* = 25)	59 (42.5; 75.6)	9.4 (4.2; 14.7)	46.8 (31.1; 62.4)	2.8 (1.3; 4.4)
UMI (*n* = 16)	74.1 (57.3; 90.8)	7.4 (4.4; 10.3)	61.8 (45.6; 78.1)	4.9 (2.1; 7.7)
UI (*n* = 41)	96.7 (79.1; 114.4)	14.6 (8; 21.1)	70.7 (55; 86.3)	11.5 (6.8; 16.1)
*p*‐value	< 0.05	< 0.05	< 0.05	< 0.05
Corr. Index	0.34	0.37	0.49	0.37

#### 3.2.1 Direct Healthcare Costs

The total direct healthcare costs are shown in Table 2. These included the cost of healthcare staff (medical assistants, doctors, nutritionists, nurses, etc.) to visit the patients during a follow‐up visit, as well as treatment costs both by the perspective of the healthcare provider and by the patient, that is OOP expenditure for drugs. The mean monthly direct healthcare costs amounted to $8.7 per patient, with treatment costs covered by the healthcare provider accounting for half (51%) of the total. Interestingly, the inverse was reported by patients with T2DM, whose treatment costs paid OOP account for 69% of the total direct healthcare costs. Overall, higher costs were incurred by patients suffering from both T2DM and HTN ($11.4), by male patients ($10), patients from rural facilities ($9.1), and those in the UI class ($14.6).

**TABLE 2:** Direct healthcare monthly costs in $ of the programme per patient by disease, sex, location, and income group broken down by type (staff costs, treatment costs by the HC system and OOP treatment costs).

Direct healthcare costs per patient per month, $ (95% CI)				
	Total costs ($)	Healthcare staff ($)	Treatment (covered by HC system) ($)	Treatment (OOP by patient) ($)
Overall	8.7 (7.5; 10)	0.5 (0.5; 0.6)	4.5 (4.3; 4.7)	3.7 (2.5; 4.9)
By disease				
T2DM (*n* = 97)	7.7 (5.2; 10.2)	0.3 (0.3; 0.3)	2.1 (2.1; 2.1)	5.3 (2.8; 7.8)
HTN (*n* = 105)	7.2 (6.2; 8.3)	0.6 (0.6; 0.7)	5 (5; 5)	1.6 (0.5; 2.6)
Both (*n* = 96)	11.4 (9; 13.7)	0.7 (0.6; 0.8)	6.3 (6.3; 6.3)	4.4 (2; 6.7)
*p*‐value	< 0.05	< 0.05	< 0.05	< 0.05
By sex				
Female (*n* = 197)	8.1 (6.8; 9.3)	0.6 (0.5; 0.6)	4.6 (4.3; 4.8)	2.9 (1.7; 4.2)
Male (*n* = 101)	10 (7.5; 12.5)	0.5 (0.4; 0.5)	4.3 (3.9; 4.7)	5.2 (2.7; 7.7)
*p*‐value	0.15	< 0.05	0.25	0.11
By location				
Rural (*n* = 138)	9.1 (7.4; 10.8)	0.6 (0.5; 0.6)	4.5 (4.2; 4.8)	4 (2.4; 5.7)
Urban (*n* = 160)	8.4 (6.7; 10.1)	0.5 (0.5; 0.6)	4.4 (4.2; 4.7)	3.4 (1.7; 5.1)
*p*‐value	0.69	0.76	0.71	0.60
By income group				
< PL (*n* = 42)	7.1 (4.7; 9.6)	0.5 (0.4; 0.6)	4.4 (4; 4.9)	2.2 (−0.2; 4.6)
LMI (*n* = 25)	9.4 (4.2; 14.7)	0.5 (0.4; 0.6)	4.9 (4.1; 5.6)	4.1 (−1.1; 9.2)
UMI (*n* = 16)	7.4 (4.4; 10.3)	0.4 (0.3; 0.5)	3.9 (3.1; 4.7)	3.1 (0.2; 5.9)
UI (*n* = 41)	14.6 (8; 21.1)	0.4 (0.3; 0.5)	3.6 (2.9; 4.3)	10.5 (4; 17.1)
*p*‐value	0.05	0.13	0.08	< 0.05
Corr. Index	0.37	0.33	0.32	0.35

In terms of OOP expenses, female patients were observed to incur almost half ($2.9) of the OOP treatment costs reportedly incurred by male patients ($5.2). While no difference was observed with regards to the treatment costs incurred by the healthcare provider between rural and urban facilities, patients in rural settings were observed to incur slightly higher OOP costs for treatment than patients from urban settings. Finally, patients in the UI class reported the highest OOP costs ($10.5), markedly higher than those in the other income categories, whose OOP costs ranged between $2.2–4.1.

#### 3.2.2 Direct Non‐Healthcare Costs

The total direct non‐healthcare costs included OOP expenditures incurred by the patient for the management of their disease, such as transportation costs to attend medical visits, as well as the cost of their diet (Table 3). The mean monthly direct non‐healthcare costs amounted to $36.9 per patient, driven by the costs incurred for maintaining a diet adapted to their disease profile ($35.9). The latter was highest in patients with T2DM ($48.9), male patients ($42), and patients from rural settings ($36.8). Finally, patients in the UI class reported sustaining almost double the OOP costs for diet ($70.7) reported by patients under the poverty line ($32.2).

**TABLE 3:** Direct non‐healthcare monthly costs in $ of the programme per patient by disease, sex, location, and income group, broken down by type (diet and transport).

Mean direct non‐healthcare costs per patient per month (95% CI)			
	Total costs ($)	Diet costs ($)	Transport costs ($)
Overall	36.9 (32.5; 41.3)	35.9 (31.6; 40.3)	1 (0.8; 1.2)
By disease			
T2DM (*n* = 97)	50.1 (41.6; 58.6)	48.9 (40.5; 57.4)	1.1 (0.8; 1.5)
HTN (*n* = 105)	24.2 (18.3; 30.1)	23.6 (17.7; 29.5)	0.6 (0.4; 0.9)
Both (*n* = 96)	37.5 (29.8; 45.3)	36.3 (28.7; 44)	1.2 (0.9; 1.6)
*p*‐value	< 0.05	< 0.05	< 0.05
By sex			
Female (*n* = 197)	33.7 (28.4; 39.1)	32.9 (27.6; 38.1)	0.9 (0.7; 1.1)
Male (*n* = 101)	43.1 (35.3; 50.9)	42 (34.3; 49.7)	1.1 (0.8; 1.5)
*p*‐value	< 0.05	< 0.05	0.29
By location			
Rural (*n* = 138)	37.7 (31.8; 43.6)	36.8 (31; 42.6)	0.9 (0.7; 1.1)
Urban (*n* = 160)	36.2 (29.8; 42.7)	35.2 (28.8; 41.6)	1 (0.7; 1.3)
*p*‐value	0.74	0.73	0.57
By income group			
< PL (*n* = 42)	33 (24.9; 41.1)	32.3 (24.2; 40.4)	0.7 (0.3; 1.1)
LMI (*n* = 25)	46.8 (31.1; 62.4)	45.5 (29.9; 61.1)	1.3 (0.6; 1.9)
UMI (*n* = 16)	61.8 (45.9; 77.7)	61.1 (44.8; 77.3)	0.8 (−0.1; 1.6)
UI (*n* = 41)	70.7 (54.7; 86.6)	68.8 (53.1; 84.4)	1.9 (1; 2.9)
*p*‐value	< 0.05	< 0.05	< 0.05
Corr. Index	0.49	0.38	0.38

#### 3.2.3 Productivity Losses

**FIGURE 3:**
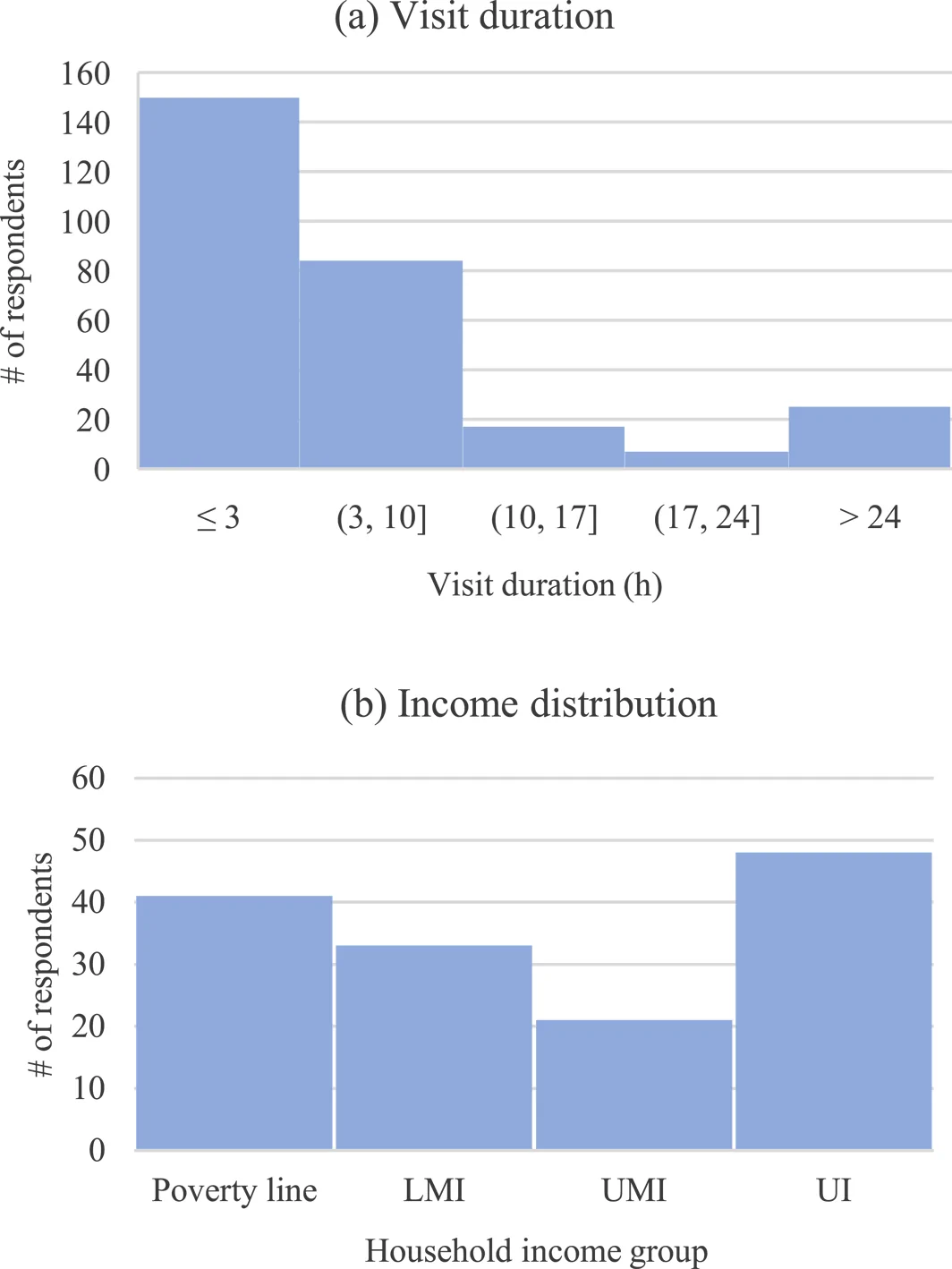
Distribution of (a) duration of visits and (b) patients across income groups in the sample of patients interviewed.

The monthly cost of patient time accounted for the loss of the patient's time from other productive activities, expressed as loss in income, to attend medical visits, including travel time, and amounted to $4.2 and was the highest for patients with T2DM or HTN (Table 1). Patients from all disease groups reported having to visit the health facility on a monthly or bi‐monthly basis, resulting in 10 visits per year on average, with each visit taking an average of 7 h out of their time (Table 4), including transportation to reach the health facility. The longest visits were reported by patients suffering from both T2DM and HTN, by males, by patients in urban facilities, and by those in the UMI class. As illustrated in Figure 3a, for half of the patients interviewed (53%) a typical visit would necessitate less than 3 h of the patient's time, while, for one‐third of the sample, this could take up to 10 h, and for the rest (18%) this could take more than 10 h, reaching even beyond 24 h.

**TABLE 4:** Productivity losses in $, duration of visits in hour and monthly income per patient by disease, sex, location, and income group.

Productivity losses, duration of visits and monthly income per patient by disease (95% CI)			
	Productivity losses ($)	Visit duration (h)	Average monthly income ($)
Overall	4.2 (2.9; 5.4)	7 (5.8; 8.2)	195.9 (162.4; 229.4)
By disease			
T2DM (*n* = 97)	4.6 (2.4; 6.8)	6.8 (5; 8.5)	272.3 (208.9; 335.7)
HTN (*n* = 105)	4.4 (1.9; 6.8)	6.7 (4.4; 9)	123 (82.7; 163.3)
Both (*n* = 96)	3.3 (1.6; 5)	7.6 (5.6; 9.6)	170.1 (115.2; 225)
*p*‐value	0.36	0.98	< 0.05
By sex			
Female (*n* = 197)	3.3 (1.7; 4.8)	6.2 (4.9; 7.6)	162 (120.4; 203.6)
Male (*n* = 101)	5.4 (3.3; 7.4)	8.4 (6.1; 10.7)	240.4 (186.4; 294.4)
*p*‐value	0.11	0.12	< 0.05
By location			
Rural (*n* = 138)	2.9 (2; 3.9)	6.2 (4.6; 7.7)	186.9 (132.9; 241)
Urban (*n* = 160)	5.1 (3; 7.1)	7.7 (6; 9.5)	202.5 (159.4; 245.6)
*p*‐value	0.06	0.80	0.59
By income group			
< PL (*n* = 42)	1 (0.5; 1.5)	7.6 (3.7; 11.5)	51.3 (34.4; 68.3)
LMI (*n* = 25)	2.8 (1.3; 4.4)	5.9 (2.9; 8.9)	101.6 (93; 110.2)
UMI (*n* = 16)	4.9 (2.1; 7.7)	12.1 (1.9; 22.3)	195.7 (174.2; 217.2)
UI (*n* = 41)	11.5 (6.8; 16.1)	6.2 (3.5; 9)	509.7 (434.7; 584.6)
*p*‐value	< 0.05	0.94	< 0.05

#### 3.2.4 Impact on Household Income

To investigate the potential impact of disease burden on equity across different income groups, we distinguished between the burden borne by the healthcare system and that borne by the patients and their families. The latter included all OOP costs for medications, diet, transport as well as productivity losses.

To evaluate the impact of these costs on the overall income, we estimated what percentage of the total patient's income is affected by the disease (Table 5). When asked about their income, only half of the sample provided an answer (*n* = 143). In our sample, one‐third was estimated to fall under the poverty line, while half of the sample reported income levels that would place them in the upper and upper‐middle income group as defined by the World Bank [49] (Figure 2b). Proportionally, the overall cost was more burdensome on patients from lower income groups, especially those below the poverty line. The average cost incurred by the lowest income group ($36.2) accounted for the majority (71%) of their reported monthly income ($51.3), with the proportion ranging from 18% to 71% across income groups (Table 5). Furthermore, 3% of the sample reported that their income is insufficient to cover the costs incurred for the management of their disease. This measure was based on respondents' self‐reported perception of financial hardship, derived from a direct survey question on whether their household income was sufficient to cover the costs of disease management, and was not calculated using a predefined catastrophic expenditure threshold.

**TABLE 5:** Average monthly costs at patient level in $, including costs for OOP drug expenditures, transport, diet and productivity losses (95% CI), average monthly income per patient in $ (95% CI) and percentage of patient income spent on disease.

	Patient costs ($)	Average monthly income per patient ($)	Percentage of patient income spent on disease
Overall	44.8 (40.1; 49.5)	195.9 (162.4; 229.4)	23%
By disease			
T2DM (*n* = 97)	60 (50.9; 69.1)	272.3 (208.9; 335.7)	22%
HTN (*n* = 105)	30.1 (23.6; 36.6)	123 (82.7; 163.3)	24%
Both (*n* = 96)	45.2 (37; 53.4)	170.1 (115.2; 225)	27%
By sex			
Female (*n* = 197)	39.9 (34.3; 45.6)	162 (120.4; 203.6)	25%
Male (*n* = 101)	53.7 (45.4; 62)	240.4 (186.4; 294.4)	22%
By location			
Rural (*n* = 138)	44.7 (38.6; 50.8)	186.9 (132.9; 241)	24%
Urban (*n* = 160)	44.7 (37.8; 51.6)	202.5 (159.4; 245.6)	22%
By income group			
< PL (*n* = 42)	36.2 (27.7; 44.7)	51.3 (34.4; 68.3)	71%
LMI (*n* = 25)	53.6 (37.1; 70.2)	101.6 (93; 110.2)	53%
UMI (*n* = 16)	69.8 (53.1; 86.5)	195.7 (174.2; 217.2)	36%
UI (*n* = 41)	92.7 (75.1; 110.3)	509.7 (434.7; 584.6)	18%

## 4 Discussion

Measuring the economic burden of NCDs in LICs is essential for policymakers to allocate resources efficiently, prioritise healthcare interventions, and develop cost‐effective strategies to combat chronic diseases. By understanding the financial strain imposed by NCDs, such as T2DM and HTN, policymakers can better advocate for international funding and support.

Our study was the first economic analysis of T2DM and HTN in Mozambique, offering novel and up‐to‐date estimates on the overall economic burden of these diseases from the healthcare provider perspective, that is the healthcare system, and from the societal one, that is costs borne by the patients. It also provided the amount and types of costs incurred for the implementation of a disease management programme and on the average cost per visit.

The total monthly cost for management of the NCDs from the healthcare system perspective, amounted to $5.30 per patient, covering healthcare personnel ($0.5), treatments ($4.5), and the average cost per visit ($3.2 per year). Cross‐country comparisons revealed that Mozambique's costs were higher than Malawi's [51], but lower than Uganda's [52], Kenya's [10], Rwanda's [53] for comparable outpatient NCD care. Additionally, the $3.20 per visit metric suggested relatively efficient service delivery compared to tertiary facilities in Nigeria [12], yet remained financially burdensome for a system where total health expenditure barely exceeds $50 annually per capita [54].

This highlights the significant strain that NCDs can place on the Mozambican healthcare system and underscores the demand for suitable and sustainable interventions for their effective management. In order to achieve the UHC goals set by international organisations, there is a need for a structural increase in government financing as well as the exploration of additional cost‐sharing mechanisms for patients, as already trialled in other Sub‐Saharan African countries, such as the introduction of universal basic health insurance [55, 56, 57]. Additionally, programs implemented by external partners are critical in helping the healthcare system respond to these increasing demands [58, 59]. Their efforts to strengthen healthcare infrastructure, provide essential training, and improve access to treatment for chronic disease patients are central in addressing the challenges posed by NCDs [60, 61].

This research not only shed light on the overall economic burden borne by the healthcare system, but also illustrated the costs borne by the patients and their effects on equity across different socioeconomic groups. More specifically, from a patient perspective, the mean total monthly costs differed significantly across the disease groups in this study, driven by differences in direct costs. While for T2DM patients, the increased cost compared to HTN patients can be accounted for by direct non‐healthcare costs, primarily for OOP on diet, for patients diagnosed with both diseases, higher costs are accounted for by direct healthcare costs, specifically cost of treatment (drugs).

The mean monthly cost for T2DM patients ($62.4) estimate was in line with those of a global systematic review of the economic costs of T2DM [35, 43, 46], which uncovered rising and higher direct than indirect costs overall, while indicating that a big part of these costs in LIC is borne by the patients through OOP expenditures [35]. The direct cost estimated for T2DM patients was also much less than those reported in Mali [39] and Nigeria [12]. The latter reported a total cost of $365 per month on average, and identified diet, drugs, and lab tests as the cost drivers, while our study estimated these costs at $50.1 per month per patient, confirming diet as the cost driver. For T2DM patients, we found an annual cost for medications of $88.8, from the combined perspective of the healthcare provider ($2.1 per month) and patient ($5.3 per month). This compares less than the annual medication costs reported by a review on T2DM costs in LMIC of $177, which were reported to be highly varied [46].

Furthermore, our estimate of the total monthly cost for HTN patients of $35.8 per patient, when reported at an annual basis ($429.6) for Mozambique, was lower than another estimate of $476.5 in Kenya [10]. The study reported that 59% of households experienced catastrophic costs at the 40% non‐food expenditure threshold, while further cost breakdowns were provided for the main cost drivers, those being the mean annual cost of $168.9 for medicines, and $57.7 for user fees. Our findings were however higher than the estimate provided by a similar study in Ethiopia of $105.6 per HTN patient per month, which in contrast observed that indirect costs accounted for more than 80% of the costs [32]. This high variability in cost estimates across different countries and cost drivers suggested that the findings were context‐dependent and indicate the need for further research.

Additionally, our findings suggested that the cost borne by the patient per income group was a function of the disposable income available, with the highest costs observed to be incurred by patients earning the highest reported incomes, and inversely, the lowest income group with the lowest available income, reporting to bear the lowest costs. Nevertheless, in relative terms, while the disease management costs in the highest income group represented approximately 18% of the income reported in that group, they represented 71% of the reported income of the group falling below the poverty line. This finding is important when considering the proportion of population living in poverty in Mozambique and the economic impact that NCDs could have in LICs. According to the United Nations Development Programme, in 2023 almost 62% of the Mozambican population was considered multi‐dimensionally poor in terms of health, standards of living, and education, in addition to the international poverty rate [49]. This would imply that chronic diseases such as T2DM and HTN could pose additional economic strain, especially on the poorer segments of the population. Nevertheless, our analysis also indicates that higher costs are concentrated in higher‐earning patients, which can be explained considering the greater capacity of such patients to respond to the financial needs of managing their chronic disease.

This study had some limitations. A major limitation was the number of patients who provided information on their income level which represented less than half of the sample. This heavily impeded the interpretation of the findings as to the analysis of the data per income group. Secondly, the primary data collection reflected perceptions reported by patients and healthcare workers themselves, resulting in recall bias. This bias was not able to be verified due to the unavailability of other sources of monitoring of costs pertaining to the chronic diseases or sample group under investigation. In addition, the assessment of catastrophic financial burden relied on a self‐reported and subjective measure of income insufficiency rather than on standardised expenditure‐based thresholds, which may limit comparability with studies using formal definitions of catastrophic healthcare expenditure. Thirdly, while balanced representation was achieved across disease groups and location, with respect to sex, females were over‐represented in the sample by two‐thirds. Furthermore, the study scope was limited to the outpatient costs and did not consider inpatient costs that may represent a portion of the cost‐of‐illness that is not negligible. Finally, the manuscript focused on the costs and drivers of costs, that is the inputs, without taking into account relative outputs and outcomes. On indirect costs, specifically, the analysis was conducted considering presenteeism (i.e., time lost from productive activities); while the survey included a question on absenteeism (i.e., whether the patient/respondent had to terminate their work due to their illness), of the respondents (*n* = 159), 44 responded yes (28%), this was not quantified or used in the analysis.

## 5 Conclusion

This study provided a first estimate of the burden of chronic diseases such as T2DM and HTN in Mozambique at both the provider and patient levels. Additionally, the study offered insight on the high economic impact of the diseases across income groups, with those living under the poverty line, who represent a significant proportion of the Mozambican population, most negatively affected.

Our results suggested that the current funding model, which is predominantly hospital‐centric or focused on infectious diseases, is insufficient to address the growing NCD epidemic. To improve sustainability, the findings underscore the urgency of reorienting government funding towards decentralised primary care.

This could be achieved through innovative delivery and contracting models, such as expanding public‐private partnerships for drug distribution or implementing performance‐based financing, but also intersectoral collaboration to integrate prevention, early detection, and affordable care delivery.

Such structural reforms are vital to translate national strategic commitments into effective service delivery, reducing the reliance on out‐of‐pocket expenditures and ensuring more equitable access to chronic care.

Future research should explore the cost‐effectiveness of preventive interventions and the potential of community‐based models to reduce barriers to access, ensuring equitable and sustainable management of NCDs in low‐resource Countries.

## Author Contributions

A.D.T., M.V., G.P., A.T.: Concept and design. M.V., S.L., P.M., F.C., C.M., C.A.: Analysis and interpretation of data. M.V., P.B., A.T.: Draughting of the manuscript. M.V., P.B., G.P., A.T., A.D.T.: Critical revision of the paper.

## Funding

This study was financed by the Italian Agency for Development Cooperation, Ministry of Foreign Affairs (AICS) under the Project titled ‘Prevenzione e controllo delle malattie non trasmissibili’, AID: 11375.

## Ethics Statement

A protocol for this study was redacted following the guidelines provided by the National Committee on Bioethics for Health of the Republic of Mozambique's Ministry of Health (CNBS) (IRB00002657) and approved by the relevant authorities in March 2023.

## Conflicts of Interest

The authors declare no conflicts of interest.

## Supporting information


Supporting Information S1

## Data Availability

The data that support the findings of this study are available on request from the corresponding author. The data are not publicly available due to privacy or ethical restrictions.
