# Supplementary material for: Exploring the Socio‐Economic Toll of Non‐Communicable Diseases in Low‐Resource Settings: Novel Evidence From Mozambique
Source: Int J Health Plann Manage. 2026 May 19;41(5):809–21. doi: 10.1002/hpm.70091 (PMC13551525; doi:10.1002/hpm.70091)
Supplement: Supplementary file 1 [file HPM-41-809-s001.docx]

**Supporting Information**

## **Supporting Information 1: Health Facilities**

The table below reports the list of all Health Facilities (HF) in which the Doctors with Africa CUAMM Program has been implemented.

| **Province** | **District** | **HF Name** | **HF Setting** |
| --- | --- | --- | --- |
|  |  |  |  |
| **Maputo** | Matola | CS Matola C | Urban |
|  | Matola | CS São Damaso | Semi-urban |
|  | Moamba | CS Moamba Sede | Semi-urban |
|  | Moamba | CS Sabie | Rural |
| **Sofala** | Beira | CS Macurungo | Urban |
|  | Beira | CS Nhaconjo | Semi-urban |
|  | Dondo | CS Mutua | Rural |
|  | Nhamatanda | Rural Hospital Nhamatanda | Hospital 2° level / Rural |
|  | Nhamatana | CS Lamego | Rural |
| **Zambezia** | Quelimane | CS 17 de Setembro | Urban |
|  | Quelimane | CS Coalane | Rural |
|  | Mocuba | CS Mocuba Sede | Rural |
|  | Mocuba | District Hospital Mocuba | Hospital 2° level / Rural |
|  | Mocuba | Mugeba | Rural |

**Supporting information 2: Average number of patient visits**

Average number of patient visits per health facility setting in the 6-month period prior to data collection.

| **HF Setting** | **Mean number of visits per month** | | |
| --- | --- | --- | --- |
|  | **T2DM** | **HTN** | **T2DM & HTN** |
| Semi-urban | 35 | 789 | 99 |
| Rural | 9 | 332 | 20 |
| Urban | 48 | 804 | 137 |
| Hospital 2° level | 20 | 337 | 59 |
| **Grand Total** | **112** | **2262** | **315** |

**Supporting information 3: Study sample size**

| **Setting** | **T2DM** | **HTN** | **T2DM & HTN** | **Total** |
| --- | --- | --- | --- | --- |
| ***Actual*** | | | | |
| Rural | 45 | 44 | 49 | 150 |
| Urban | 52 | 61 | 47 | 153 |
| **Total** | **97** | **105** | **96** | **298** |

## **Supporting Information 4: Patient pathway**

The analysis of OOP expenditures the patient must cover as well as the time spent during the visit to the clinic by patients, caregivers and healthcare personnel during the patient pathway was based on information gathered through interviews during primary data collection. A description of a standard patient visit is described in Box 1.

Box 1: Patient visit for T2DM and HTN patients following the CUAMM program

1. Measurement of blood pressure in the dedicated corner, regardless of whether the patient is followed or not in the NCDs clinic.
2. If a blood pressure ≥ 140/90 is suspected, the measurement is repeated after 30 minutes:
   a. If the hypertension persists in the classification of mild to moderate (according to the table used in the *cantinho*), the weight and height are measured and the patient is sent to consult the NCDs with a sheet in which the pressure is noted, the measurements taken and the date.
   b. If the blood pressure is elevated (severe hypertension), the patient is referred to the emergency department.
   c. If the patient shows up at the next NCDs appointment they are referred to the NCDs outpatient appointment. The data is recorded in the daily screening form.
3. Patients who come to the Diabetes Numeracy Test consultation, if they are in the follow-up phase, deliver their user card in which the NID (identification code) appears, which facilitates the search for their file and the measurement of the Arterial Test is repeated. The search of the medical records is carried out by an administrator, if present, or by an activist. Otherwise, the user data is logged and a new patient record is opened.
4. Suspicious patients go first for the visit for which they came to the HF (reason for the visit) and then, if there is time, or on another day, they go to the NCDs outpatient clinic.
5. The doctor evaluates the patient, fills in the consultation card, the patient's medical record (follow-up card, clinical diary) and makes recommendations for the patient, whether it is medication, laboratory test prescription and indicates the date of the next appointment, which the accompanying staff (auxiliary staff) fills in the diary and in the patient file.
6. Finally, the patient goes to the pharmacy to collect the prescribed drugs.

## **Supporting Information 5: Patient characteristics**

In total, 303 responses were collected. Table S2.1 provides an overview of the respondent demographic characteristics. A balanced number of responses was received across the three disease groups, as presented in Table 3, as well as between rural and urban locations. Notably, more female (n=202) respondents were interviewed than men (n=101).

Table S2.1: Patient characteristics (age, year of diagnosis, education) by disease, sex and location. Results are reported as means including uncertainty at the 95% Confidence Interval (CI) level. *The mode is used for the time of diagnosis.

|  | **Mean (95% CI)** | | |
| --- | --- | --- | --- |
|  | **Age (Years)** | **Time of diagnosis (Year)*** | **Level of education (Years)** |
| **Overall** | **56 (54;57)** | **2020** | **6 (5;7)** |
| **By disease** |  |  |  |
| T2DM (n=97) | 51 (48;53) | 2020 | 8 (7;9) |
| HTN (n=108) | 58 (55;60) | 2022 | 4 (3;5) |
| Both (n=98) | 59 (57;61) | 2020 | 6 (5;7) |
| **By Sex** |  |  |  |
| Female (n=202) | 55 (53;57) | 2020 | 5 (5;6) |
| Male (n=101) | 58 (55;60) | 2020 | 8 (7;9) |
| **By Location** |  |  |  |
| Rural (n=150) | 56 (54;58) | 2020 | 6 (5;6) |
| Urban (n=153) | 56 (54;58) | 2022 | 7 (6;7) |
| **By Income Class** |  |  |  |
| <PL (n=42) | 56 (52;61) | 2018 | 5 (4;6) |
| LMI (n=25) | 56 (51;62) | 2017 | 6 (5;8) |
| UMI (n=16) | 50 (44;55) | 2018 | 10 (9;12) |
| UI (n=41) | 51 (48;54) | 2019 | 12 (10;13) |

The average age of respondents for each disease ranged from 51-59 years old. Most patients received their diagnosis in 2020, the first year of implementation of the CUAMM program, except for HTN patients and patients from urban settings who responded most frequently to have been diagnosed in 2022.

The average number of years of education completed was reported at 6 years (95% Confidence Interval – CI: 5;7), with T2DM patients reporting the highest number of years in education (8 years) and HTN patients the lowest by a half (4 years). The distribution of the number of years in education reported in the sample is shown in Figure 1a. In more detail, female patients and patients from rural settings were found to have spent an average of approximately 5 and 6 years in education, respectively, whereas males and patients from urban settings had spent approximately 8 and 7 years in education, respectively (Table S2.1).

The distribution of years in education and monthly income is plotted in Figure S2.1, while the years of education per age group is shown in Figure S2.2.

Figure S2.1: Distribution of monthly income as reported by patients in the sample.

Figure S2.2: Distribution of years in education per age group of the patients in the sample.

Years of education

| Age group | Total |
| --- | --- |
| <25y (n=3) | 11.3333 |
| 25-40y (n=30) | 9.66667 |
| 41-65y (n=199) | 6.29442 |
| >65y (n=63) | 3.55738 |

Finally, respondents were asked whether they incurred insurance costs, to which only 6.6% of respondents indicated that they have an insurance, most of whom were male patients suffering from T2DM in urban settings. The insurance costs are not included in the overall cost calculation presented above due to the small number of responses (n=6 out of 300). After exclusion of an outlier value, insurance costs were calculated as $22.5 (-6.2;51.3) on average monthly.

Figure S2.3: Distribution of total OOP monthly expenditure in the sample in USD

Table S2.2: Correlation matrix between monthly income, age, education, medical visits, OOP and total cost (* denotes significance at the p<0.05 level)

|  | ***n*** | **Mean** | **SD** | **1** | **2** | **3** | **4** | **5** | **6** |
| --- | --- | --- | --- | --- | --- | --- | --- | --- | --- |
| **1. Monthly income (USD)** | 148 | 196 | 207 | - |  |  |  |  |  |
| **2. Age (years)** | 295 | 56 | 13 | −0.18* | - |  |  |  |  |
| **3. Education (years)** | 294 | 6 | 5 | 0.61* | −0.47* | - |  |  |  |
| **4. Medical visits per month** | 298 | 1 | 0 | −0.01 | −0.14 | −0.05 | - |  |  |
| **5. OOP (USD)** | 297 | 42 | 43 | 0.45* | −0.18* | 0.40* | 0.19* | - |  |
| **6. Total cost (USD)** | 298 | 48 | 44 | 0.49* | −0.18* | 0.42* | 0.20* | 0.99* | - |
